# Supplementary material for: A Medicago truncatula Autoregulation of Nodulation Mutant Transcriptome Analysis Reveals Disruption of the SUNN Pathway Causes Constitutive Expression Changes in Some Genes, but Overall Response to Rhizobia Resembles Wild-Type, Including Induction of TML1 and TML2
Source: Curr Issues Mol Biol. 2023 May 27;45(6):4612–31. doi: 10.3390/cimb45060293 (PMC10297080; doi:10.3390/cimb45060293)
Supplement: Supplementary file 1 [file cimb-45-00293-s001.zip › Supplemental Figures combined.pptx]

## Slide 1
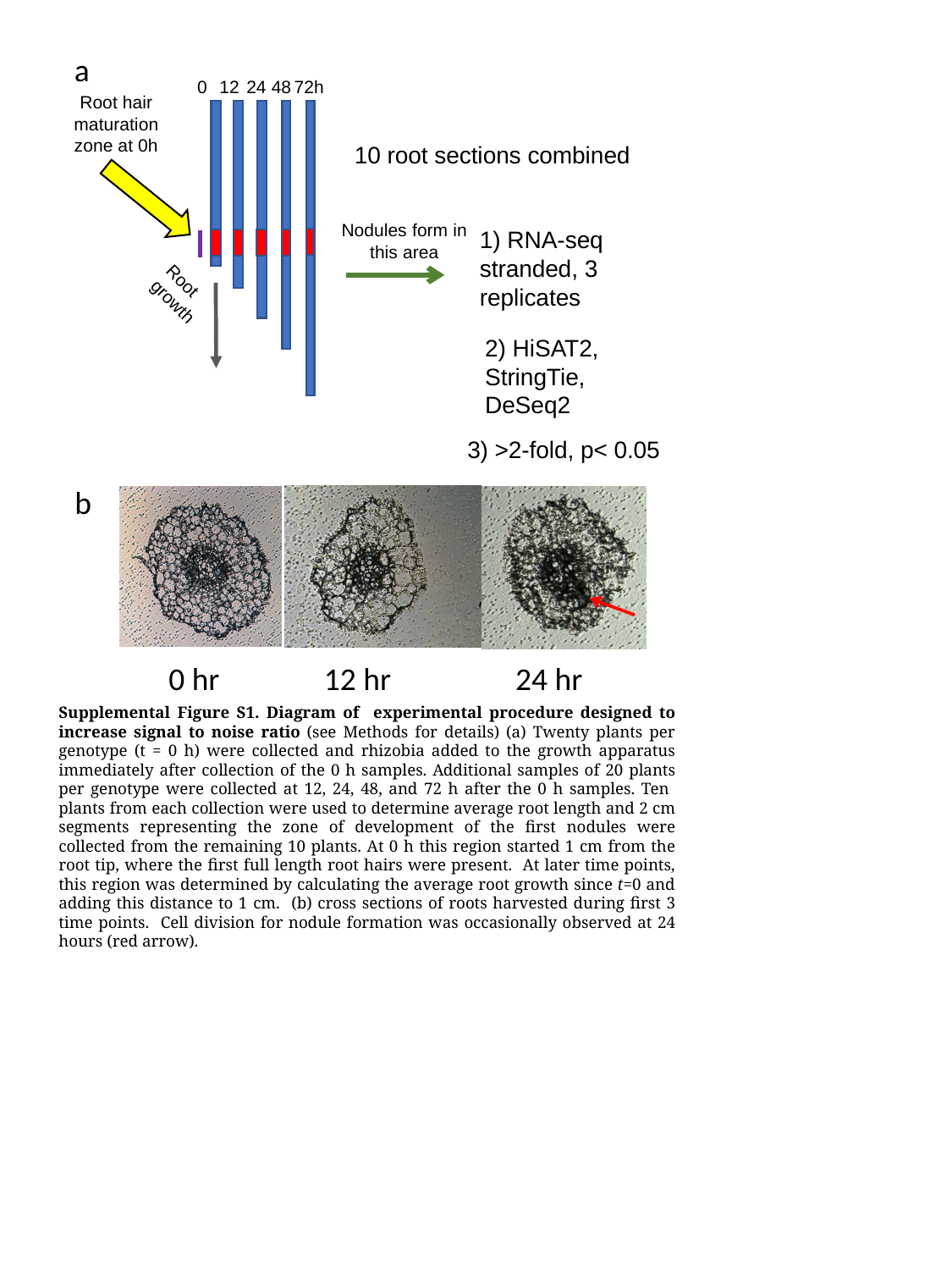

a
0
12
24
48
72h
Root hair maturation zone at 0h
10 root sections combined
Nodules form in this area
1) RNA-seq stranded, 3 replicates
Root growth
2) HiSAT2, StringTie, DeSeq2
3) >2-fold, p< 0.05
b
0 hr
12 hr
24 hr
Supplemental Figure S1. Diagram of experimental procedure designed to increase signal to noise ratio (see Methods for details) (a) Twenty plants per genotype (t = 0 h) were collected and rhizobia added to the growth apparatus immediately after collection of the 0 h samples. Additional samples of 20 plants per genotype were collected at 12, 24, 48, and 72 h after the 0 h samples. Ten plants from each collection were used to determine average root length and 2 cm segments representing the zone of development of the first nodules were collected from the remaining 10 plants. At 0 h this region started 1 cm from the root tip, where the first full length root hairs were present. At later time points, this region was determined by calculating the average root growth since t=0 and adding this distance to 1 cm. (b) cross sections of roots harvested during first 3 time points. Cell division for nodule formation was occasionally observed at 24 hours (red arrow).

## Slide 2
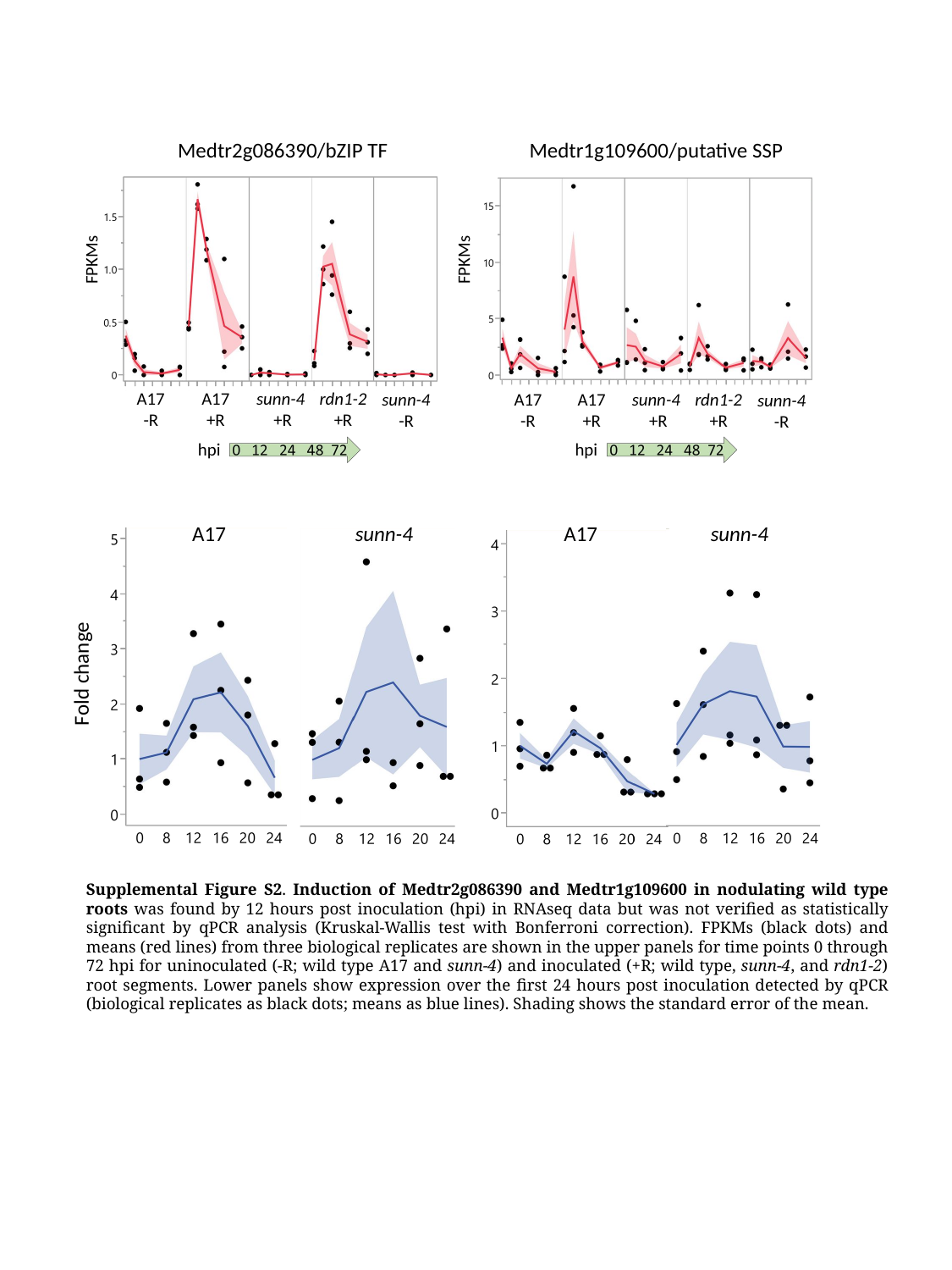

Medtr2g086390/bZIP TF
Medtr1g109600/putative SSP
FPKMs
FPKMs
A17
-R
A17
+R
sunn-4
 +R
rdn1-2
+R
A17
-R
A17
+R
sunn-4
 +R
rdn1-2
+R
sunn-4
-R
sunn-4
-R
hpi
hpi
A17
sunn-4
A17
 sunn-4
Fold change
Supplemental Figure S2. Induction of Medtr2g086390 and Medtr1g109600 in nodulating wild type roots was found by 12 hours post inoculation (hpi) in RNAseq data but was not verified as statistically significant by qPCR analysis (Kruskal-Wallis test with Bonferroni correction). FPKMs (black dots) and means (red lines) from three biological replicates are shown in the upper panels for time points 0 through 72 hpi for uninoculated (-R; wild type A17 and sunn-4) and inoculated (+R; wild type, sunn-4, and rdn1-2) root segments. Lower panels show expression over the first 24 hours post inoculation detected by qPCR (biological replicates as black dots; means as blue lines). Shading shows the standard error of the mean.

## Slide 3
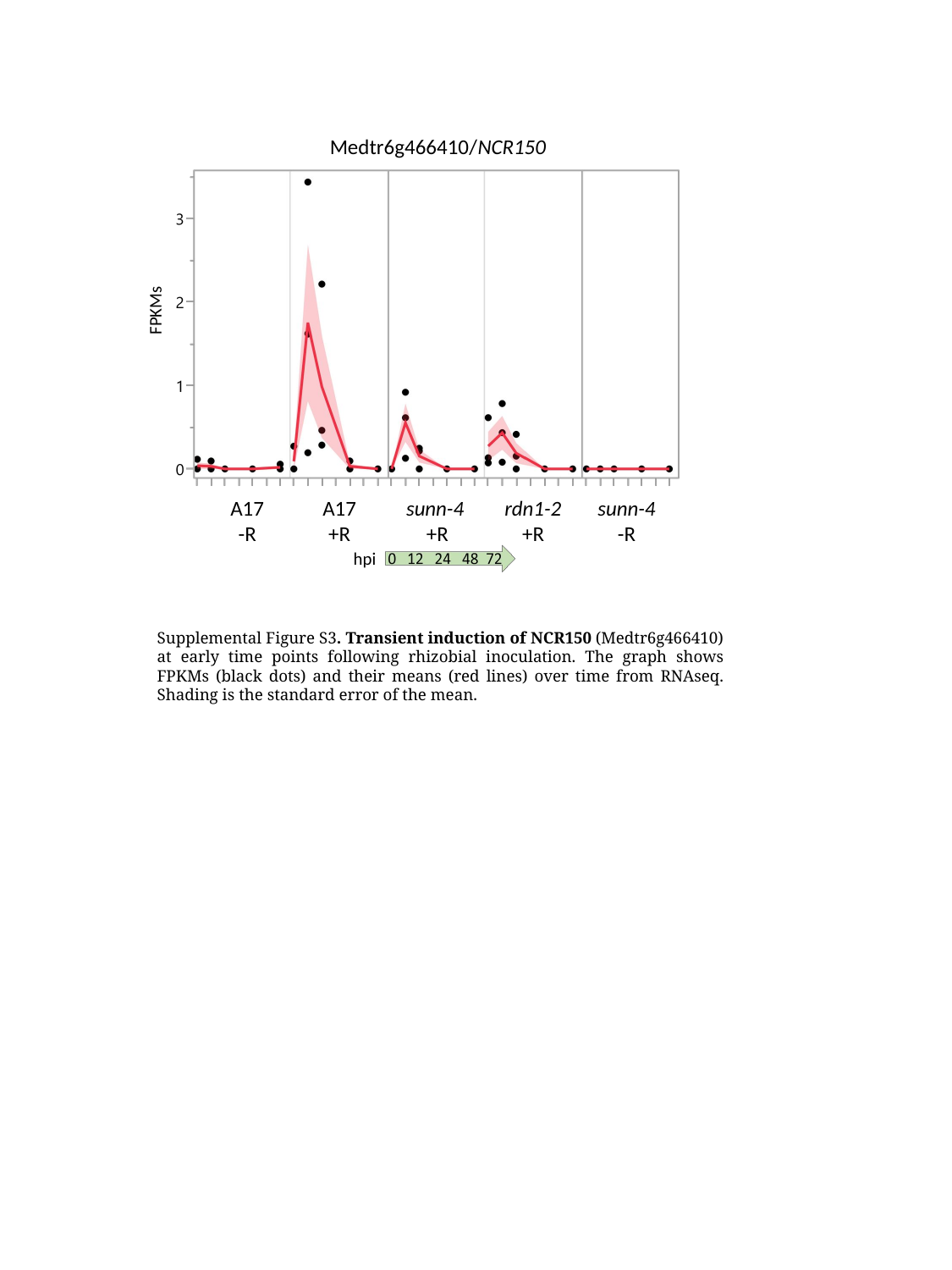

Medtr6g466410/NCR150
FPKMs
A17
-R
A17
+R
sunn-4
 +R
rdn1-2
+R
sunn-4
-R
hpi
Supplemental Figure S3. Transient induction of NCR150 (Medtr6g466410) at early time points following rhizobial inoculation. The graph shows FPKMs (black dots) and their means (red lines) over time from RNAseq. Shading is the standard error of the mean.

## Slide 4
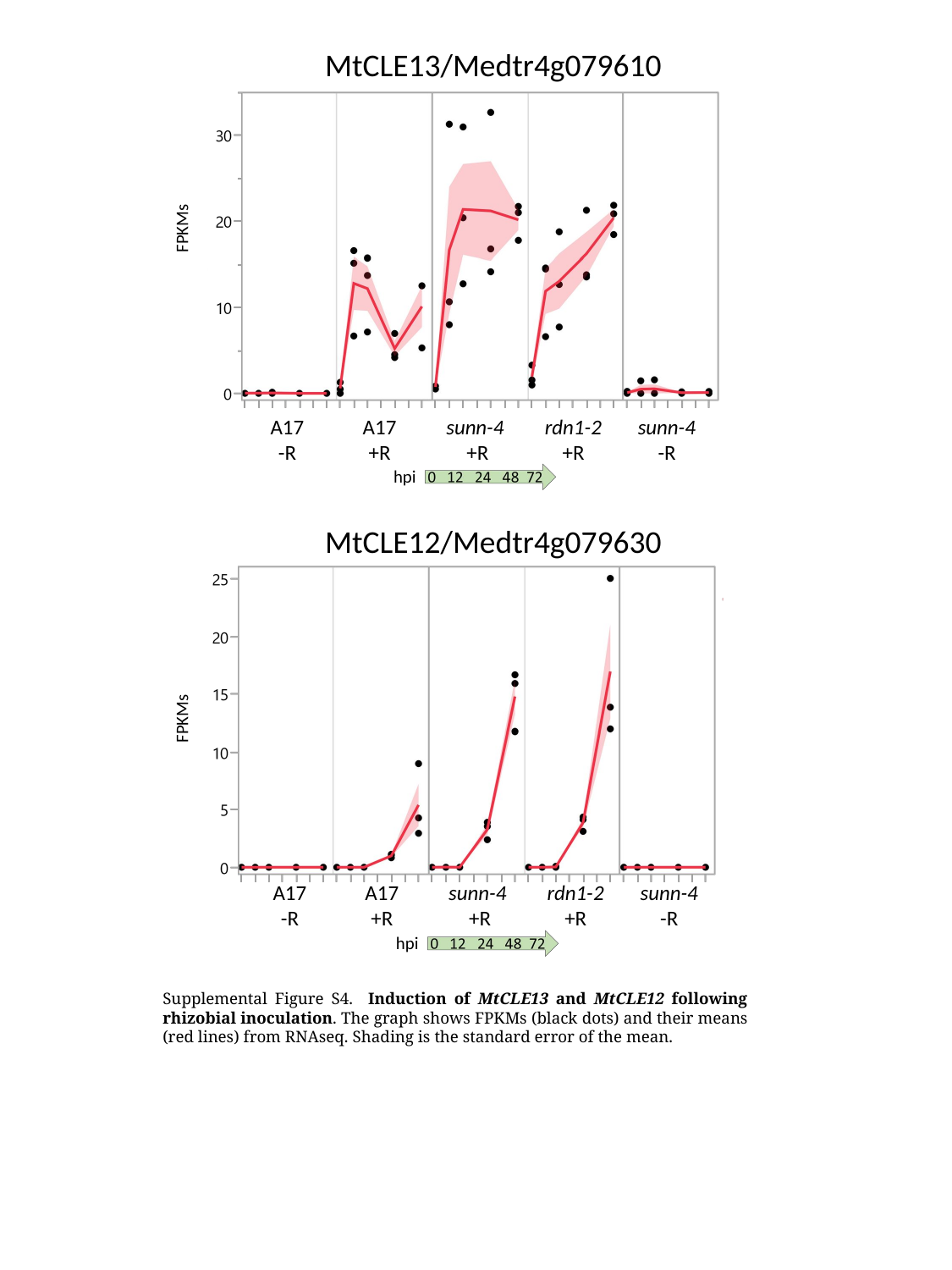

MtCLE13/Medtr4g079610
FPKMs
A17
-R
A17
+R
sunn-4
 +R
rdn1-2
+R
sunn-4
-R
hpi
MtCLE12/Medtr4g079630
FPKMs
A17
-R
A17
+R
sunn-4
 +R
rdn1-2
+R
sunn-4
-R
hpi
Supplemental Figure S4. Induction of MtCLE13 and MtCLE12 following rhizobial inoculation. The graph shows FPKMs (black dots) and their means (red lines) from RNAseq. Shading is the standard error of the mean.
